# The *Guanxi* mediating role linking organizational justice to contextual performance with age as a moderator

**DOI:** 10.1002/pchj.761

**Published:** 2024-07-24

**Authors:** Lei Tan, Yi Guan, Guojun Sheng

**Affiliations:** ^1^ College of Information and Business Management Dalian Neusoft University of Information Dalian China

**Keywords:** ageing, China, contextual performance, generational differences, *Guanxi*

## Abstract

*Guanxi*, a distinctive Chinese concept, reflects a shared vision of relationships and connections that include mutual understanding, trust, and a deep bond between individuals. Recognized for its potency in shaping the relationships that facilitate business undertakings and access to key resources, *Guanxi* is postulated as a potential mediator in the nexus between organizational justice and contextual work performance. The depth of *Guanxi*, intertwined with Chinese culture and values, may be perceived differently across age groups. Specifically, as Chinese millennials usually interact with global paradigms, generational disparities might emerge in valuing these traditional constructs. This study delves into how the dimensions of *Guanxi*—*Ganqing* (emotional connection), *Renqing* (reciprocity), and *Xinren* (loyalty)—mediate the relationship between organizational justice and contextual work performance, with chronological age as a moderator. The present study includes a convenience sample of 630 Chinese employees, aged 22–67 years, who participated in a quantitative online survey. The findings endorse the mediation role of *Guanxi*. The total influence of justice was found to be significant, as well as the indirect impacts, that were statistically salient. Although the age‐moderated mediation was not wholly substantiated, the age‐specific indirect effects of *Renqing* and *Xinren* did present significant variances between millennials and those above 42 years. The relevance of this study extends beyond the academic field, shedding light on the cultural dynamics at play within Chinese organizational settings. By unveiling the relationships between *Guanxi*, organizational justice, and performance, and by elucidating the age‐specific variations therein, this research provides insights for organizational leaders and human resource professionals. Based on these findings, businesses can craft targeted interventions that capitalize on the strengths of *Guanxi*, ensuring fair practices and enhancing performance across diverse age groups. Further, recognizing the unique attributes and values of different generational cohorts can aid in fostering a harmonious, culturally attuned, and efficient workplace environment.

## INTRODUCTION

In the evolving landscape of organizational studies, understanding employee performance and engagement antecedents within distinct cultural contexts remains relevant (Chen et al., 2023; Sheikh & Jamshed, 2023). The examination of workplace behaviors, and more specifically, contextual performance, has seen a renewed focus in the academic world, largely attributed to its significance in facilitating that organizations could reach their objectives (Boccoli et al., 2023). While a portion of this exploration has been centered on Western paradigms, there is an increasing recognition of the need to understand such phenomena in diverse cultural settings, specifically in China (Han et al., 2023).

China's mix of rich historical traditions and contemporary organizational practices offers insights for researchers about the understanding of how traditional Chinese values, deeply embedded in the ethos of the Chinese workplace, influence modern organizational behaviors (Dobrucalı, 2020). Concepts like *Guanxi*, a foundational principle in Chinese interpersonal relationships, have implications in this context, potentially shaping how Chinese workers perceive fairness and how they perform beyond their stipulated job roles. In this line, “contextual performance” has been defined as a type of employee behavior that, not being part of their specified tasks, has the potential to contribute to the organizational objectives (Banwo & Du, 2020). By fostering workplace behaviors that emanate from a sense of justice and strong interpersonal relationships, organizations can encourage ethical business practices, social responsibility, and even environmental stewardship. This is especially pertinent for China, as it seeks to balance rapid economic growth with long‐term sustainability goals. Furthermore, the dynamic brought about by generational differences adds another layer of complexity (J. Li, Huang, et al., 2023). With millennials interacting between global influences and traditional Chinese values, and older workers potentially adhering more closely to traditional tenets, the moderating role of age in relation to *Guanxi* dimensions and contextual performance is proposed (L. Liu & Jia, 2021).

This study aims to test the relationship between organizational justice and contextual performance, mediated by *Guanxi* dimensions, as well as the potential moderating role of age. By juxtaposing millennials against older workers, we hope to highlight the differences of how these groups perceive and enact *Guanxi* in their workplace behaviors. Through this investigation, we try to expand the understanding of organizational behavior in the Chinese context, providing insights for both academicians and practitioners.

## THEORETICAL FRAMEWORK AND HYPOTHESES DEVELOPMENT

### Contextual performance among Chinese workers

Performance in the workplace is typically evaluated based on the outcomes and behaviors that directly relate to an individual's specific job tasks (Viswesvaran & Ones, 2000). However, there is another facet to performance that has garnered attention in organizational literature, termed “contextual performance.” This refers to those activities and behaviors that support the organizational environment, allowing the technical core to do its job but not a part of the core itself (Borman & Motowidlo, 1993). These behaviors, while not directly contributing to an individual's primary job tasks, play a role in organizational effectiveness (Banwo & Du, 2020).

China provides a backdrop for the study of contextual performance. The traditional Chinese workplace has historically been shaped by Confucian values, emphasizing loyalty, respect for authority, and the importance of harmonious relationships (J. Li, 2008). These values are intrinsically linked to behaviors that characterize contextual performance, such as cooperating with others, assisting colleagues, and demonstrating patience and adaptability (Zhou et al., 2017).

At the heart of Chinese cultural values is the principle of collectivism, which contrasts with individualistic orientations prevalent in many Western societies. Collectivism prioritizes group goals over individual ambitions and places a significant emphasis on harmony and interdependence among group members (Hofstede, 2001). These values could impact on the workplace (M. Li, Li, et al., 2023). According to Chen and Tjosvold (2007), Chinese employees tend to engage more in cooperative behaviors, aimed at ensuring team harmony and achieving collective objectives (Barbalet, 2023). Such behaviors characterize contextual performance, where actions are intended to benefit the organization and its members holistically, rather than just serving individual ends. This collectivist inclination can directly promote contextual performance among Chinese workers. The emphasis on maintaining group harmony and cohesion necessitates behaviors oriented to the organizational environment (Sun et al., 2023). For example, assisting peers or volunteering for additional responsibilities can be seen as being oriented towards group success and harmony (Chen et al., 2013).

In summary, the concept of contextual performance, aligned with collectivism and the traditional Confucian values that prioritize group harmony and cohesion (Yu et al., 2015), seems relevant in the Chinese organizational context (L. Liu & Zhai, 2023). Understanding these cultural dynamics could be beneficial for multinational companies operating in China and for domestic firms aiming to maximize employee performance.

### Organizational justice and its impact on workers' outcomes

“Organizational justice” is a term that refers to employees' perceptions of fairness within their workplace. It encompasses the ways in which employees deem organizational decisions, policies, and actions to be fair or unfair (Greenberg, 1987, 1990). While organizational justice has been categorized into various types (Adamovic, 2023), including distributive (fairness concerning outcomes or distributions), procedural (fairness regarding the processes leading to decisions), and interactional justice (fairness in interpersonal interactions), researchers often treat organizational justice as a single, unified construct (Colquitt et al., 2001). This perspective underscores the general sense of fairness as a holistic measure, rather than expanding into specific components.

A substantial body of research has highlighted the effects of perceived organizational justice on workers' outcomes (Colquitt et al., 2023). The perception of fairness or the lack thereof has been shown to be an antecedent of job satisfaction, commitment, trust, and turnover intentions (Folger & Konovsky, 1989; Moorman, 1991).

One of the most salient outcomes influenced by perceptions of justice is employee performance. Employees who perceive their organization as just are more likely to display higher levels of job performance, as well as other positive outcomes (Eib & Cropanzano, 2023). Such workers feel valued and recognized, leading to increased motivation and engagement (Niehoff & Moorman, 1993). Conversely, perceptions of injustice can lead to reduced commitment to job tasks, increased absenteeism, and even counterproductive work behaviors (Ambrose et al., 2002). In essence, organizational justice has several implications on worker outcomes, especially performance (Gu et al., 2020).

Based on the above reviewed literature, the following hypothesis has been proposed:

Organizational justice directly and positively impacts on workers' contextual performance.

### *Guanxi* dimensions, *Ganqing* (emotional connection), *Renqing* (reciprocity), and *Xinren* (loyalty)

*Guanxi*, a term deeply rooted in Chinese culture, refers to a system of social networks and influential relationships that facilitate business and other dealings (Yang, 2016). Central to *Guanxi* are its integral dimensions: *Ganqing* represents the depth of the emotional connection in a relationship, encompassing feelings and sentiments (Chen & Chen, 2004). *Renqing*, commonly translated as reciprocity, underscores the societal norms that necessitate returning favors and maintaining a balance in the give‐and‐take of professional and personal interactions (Hwang, 1987). *Xinren* is about loyalty and trustworthiness, and it plays a pivotal role in the stability and longevity of the relationships (Chen & Chen, 2004).

Deeply entwined with Confucian values, *Guanxi* has for centuries underscored the essence of relationships in Chinese society. It aligns with Confucian tenets that emphasize the importance of interpersonal relationships and moral integrity (Guo et al., 2020; Ma & Zhang, 2022). Due to this, *Guanxi*'s principles are not mere strategies for business dealings but mirror a society's fundamental values and norms (Wu et al., 2023). In the realm of business, the weight of *Guanxi* cannot be understated. Recognized for its potency in shaping influential relationships that facilitate business endeavors and grant access to critical resources, *Guanxi* has been integral to successful enterprise in China (Ma & Zhang, 2022).

### Mediating role of *Guanxi* in the relationships between organizational justice and contextual performance

Organizational justice, denoting employees' perceptions of fairness in the workplace, has direct implications on various outcomes, including contextual performance (Faeq & Ismael, 2022). In the Chinese context, where *Guanxi* is pervasive, the bridge between organizational justice and performance may very well be paved by *Guanxi* (Ameyaw et al., 2022).

Mutual influences between justice perception and *Guanxi* in the workplace can be proposed. On the one hand, if employees feel that they are fairly treated and respected by the organization, these perceptions intensify the emotional connection with the organization, the sense of belonging and trust, as well as the call for reciprocity (Ciampa, 2023). On the other hand, if an employee perceives that the organization honors the principles of *Ganqing*, *Renqing*, and *Xinren*, their sense of justice is amplified, potentially leading to enhanced contextual performance. The trust (*Xinren*), emotional connection (*Ganqing*), and balance of reciprocity (*Renqing*) create an environment where employees not only feel valued but are also motivated to contribute beyond their defined roles (Yen et al., 2011).

The intricate web of *Guanxi*, with its cultural roots and business implications, offers an opportunity to understand the complexity between organizational justice and contextual performance. Understanding this mediating role can provide critical insights for businesses aiming to maximize their human capital's potential within the Chinese cultural context.

Based on the above reviewed literature, the following hypothesis has been proposed:

*Guanxi* dimensions—*Ganqing* (emotional connection; H2a), *Renqing* (reciprocity; H2b), and *Xinren* (loyalty; H2c)—will mediate the relationships between organizational justice and workers' contextual performance.

### Millennials versus aged workers: The moderating role of age in the relationships between *Guanxi* dimensions and contextual performance

*Guanxi*, with its deep roots in Chinese culture, signifies a system of social networks and influential relationships aiding in facilitating business and other ventures (Yang, 2016). Integral to *Guanxi* are its essential dimensions, such as *Ganqing* (emotional connection), *Renqing* (reciprocity), and *Xinren* (loyalty). These aspects, historically, have played a role in how workers perceive and understand their workplace environment and relationships (Bian, 2018).

With the influences of globalization and its consequential cultural exchange, some traditional constructs like *Guanxi* are perceived differentially across generations. While older generations might be deeply embedded in the conventional understanding of *Guanxi*, the younger, particularly the millennial generation (Generation Y, individuals born between 1981 and 1996. As of 2023, millennials would be between the ages of 27 and 42), who are part of a more interconnected and globalized world, have exhibited differentiated values and perspectives (Au, 2022; Zhou et al., 2007).

In essence, millennials, influenced by global paradigms, could perceive and engage with *Guanxi* differently than their older counterparts. This is not to suggest an abandonment of traditional values, but a more complex interpretation influenced by both global and local factors (Eckhardt & Bengtsson, 2010; Guo et al., 2020). Related research showed that age could be associated to temporal orientation, a cognitive–motivational construct that influences performance in several spheres, such as school or work (Y. Liu, Gong, et al., 2023). As information from Western societies invades China, the local population travels abroad, international educational experiences and exchanges increase, and rural citizenships move to urban environments, perceptions of traditional values and social networks could differ as a function of age. Hence, given the strong links between *Guanxi* and collectivist approaches, differences in the exhibited performance could be proposed when comparing more traditional employees (aged) with millennials.

Contextual performance, denoting the non‐task‐related contributions of employees, has been related to *Guanxi* dimensions in the Chinese workplace (Boccoli et al., 2023; Farid et al., 2023). With the age‐based differential perceptions of *Guanxi*, it is plausible to propose age as a significant moderator in this relationship. While older workers might lean heavily on traditional *Guanxi* dimensions, shaping their contextual performance largely around these values, millennials might exhibit a more blended approach, incorporating global ethical and relational perspectives (Inglehart & Baker, 2000).

As the Chinese workforce evolves, understanding generational differences, especially concerning traditional constructs like *Guanxi*, becomes relevant. Age, serving as a potential moderator, can significantly impact the dynamic interplay between *Guanxi* dimensions and contextual performance.

Based on the above reviewed literature, the following hypothesis has been proposed:

Workers' chronological age will moderate the relationships between organizational justice and their contextual performance, mediated by *Guanxi* dimensions—*Ganqing* (emotional connection; H3a), *Renqing* (reciprocity; H3b), and *Xinren* (loyalty; H3c). In addition, the magnitude of the relationships hypothesized in H2 is expected to be altered by workers' age, being greater in older workers than in millennials for *Ganqing*, *Renqing*, and *Xinren*.

**FIGURE 1:**
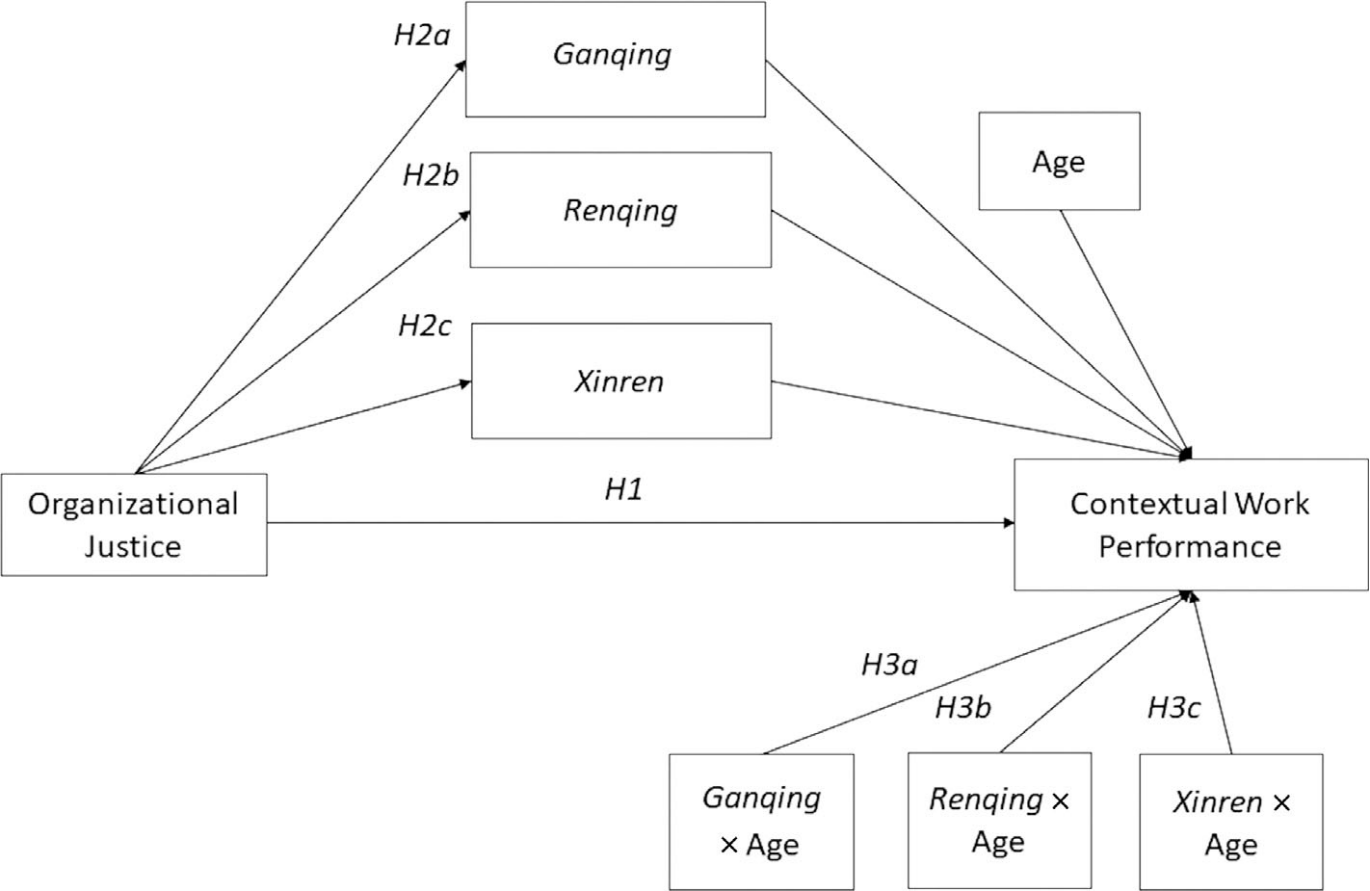
Research model with hypotheses for the parallel moderated mediation of *Guanxi* dimensions on contextual work performance.

A model depicting the research proposal is displayed in Figure 1.

## METHOD

### Participants and procedure

The sample for the present study comprised 630 Chinese employees. The age of participants ranged from 22 years and above, ensuring a mix of both millennials and older age groups. Regarding gender, among millennials (*n* = 168), 76 were females; and among works aged above 42 years (N = 463), 238 were females. Regarding educational level, 53.8% had completed high school or below, while 43.5% had a college degree, and 2.7% had a Master or Doctorial degree. Participants hailed from a diverse array of sectors, including but not limited to manufacturing (43.6%), services (23.5%), technology (10.9%), and finance (12%), as well as other sectors (10%).

Data were collected using an online survey methodology during the time‐frame between 10 and 28 February 2023. An electronic questionnaire was created, ensuring questions were clear, comprehensible, and relevant to the objectives of the study. Prior to dissemination, the survey underwent a pilot test among a small group of participants to validate its clarity and relevance.

Participants were approached through various online channels, such as professional networking sites, corporate email lists, and digital forums dedicated to Chinese professionals. They were informed about the purpose of the study, assured of the confidentiality of their responses, and were provided with the necessary details on how to complete and submit the survey. The use of an online survey also ensured flexibility for the participants, as they could answer it at a time and place convenient to them.

On accessing the survey link, participants were greeted with a brief introduction about the study and its objectives, ensuring transparency. They were also informed of their right to withdraw from the survey at any point should they choose to. Informed consent was obtained digitally before the commencement of the survey, guaranteeing their anonymity and confidentiality throughout the process.

The responses received (*N* = 645) were checked and those having incomplete answers were excluded.

### Instruments

#### Organizational justice

The Organizational Justice Scale (Colquitt et al., 2001) was employed. This scale comprises 20 items. Respondents indicated their agreement using a 5‐point Likert scale, ranging from 1 (*Strongly Agree*) to 7 (*Strongly Disagree*). Sample items include: “Do your rewards reflect the effort you have put into your work?,” “Have you been treated with respect?,” and “Has your supervisor been truthful with you?” In the present study, reliability for the global scale was adequate for Cronbach's alpha (*α* = .932, 95% CI [.925; .940]).

#### Guanxi

The *Guanxi* variable was assessed with the *Ganqing*, *Renqing*, and *Xinren*‐2 (GRX‐2) Scale (Yen et al., 2011). This consisted of four items for *Ganqing* and *Renqing* and the remaining three items for *Xinren*. In the present study, reliability for the global scale was adequate for Cronbach's alpha (*α* = .871, 95% CI [.854; .885]). *Guanxi* dimensions' reliability was tested: *Ganqing* showed adequate values both for Cronbach's alpha (*α* = .883), as well as *Renqing* (*α* = .817), and *Xinren* (*α* = .941). Examples of items are: “My (colleague‐coworker) and I are able to talk openly as friends,” “I feel a sense of obligation to this (colleague‐coworker) for doing him/her a favor,” and “This (colleague‐coworker) is trustworthy.”

#### Contextual performance

The Individual Work Performance (IWP) Questionnaire was used (Koopmans et al., 2012) and 16 items from the contextual performance subscale were employed, for example, “I have been able to fulfill my responsibilities.” In the original study (Koopmans et al., 2014), the reliability of the performance measure was *α* = .79 for the contextual performance dimension. In the current study, the reliability was for Cronbach's alpha (*α* = .802, 95% CI [.775; .826]).

#### Chronological age

Chronological age: this variable has been assessed as number of years. The variable has been categorized as millennials (aged below 42 years) and aged workers (above 42 years).

### Data analyses

Data were analyzed using JASP Version 0.17.3. PROCESS 4.2 Macro (Hayes, 2022) was used for examining moderated mediation. Initially, we tested a mediational analysis using JASP where *Guanxi* dimensions (*Ganqing*, *Renqing*, and *Xinren*) mediated the link between organizational justice and workers' contextual performance. All three dimensions of *Guanxi* were entered at the same time, testing for a parallel mediation. Finally, we used Model 14 to test the moderated mediation, with chronological age moderating effect in the relationships between *Guanxi* dimensions (*Ganqing*, *Renqing*, and *Xinren*) and workers' contextual performance. Both the mediation hypotheses and the moderated mediation hypotheses are supported when zero is not included in the 95% bias‐corrected confidence interval, and it may be concluded that the parameter is significantly different from zero at *p* < .05. Moreover, regarding moderated mediation, it was expected that the mediation process would vary in line with the different values taken by the moderating variable. This procedure was based on 5000 bootstrap re‐samples and provided a moderated mediation index, as well as estimates of the indirect effect and associated confidence intervals conditional on the specific levels of the moderator, categorized as Millennials (aged under 42 years) and aged workers (above 42 years).

## RESULTS

### Confirmatory factor analyses for the *Guanxin* Scale

The Kaiser–Meyer–Olkin = 0.830, and Bartlett's sphericity test (*χ* ^2^ = 4200.91, *df* = 45, *p <* .001) showed adequate values. The model with only one factor showed unacceptable values (*χ* ^2^ = 1865.24, *df* = 35, *p* < .001, root‐mean‐square error of approximation = .287, standardized root‐mean residual = .198). The model with three related factors showed better fit (*χ* ^2^ = 138.018, *df* = 32, *p <* .001). The average extracted variance was *Guanxi* dimensions, *Ganqing* (0.655), *Renqing* (0.600), and *Xinren* (0.847). The factorial loadings showed adequate values for all the factors, as Table 1 showed. Following the proposal of the original study (Yen et al., 2011), a second‐order factor model was tested. The three dimensions showed adequate factor loadings, being statistically significant, as can be seen in Table 2.

**TABLE 1:** Factor loadings of *Guanxi* items.

						95% confidence interval	
Factor	Indicator	Estimate	*SE*	*z*‐value	*p*	Lower	Upper
*Ganqing*	Item 1	0.601	0.033	18.256	<.001	0.536	0.665
	Item 2	0.616	0.037	16.796	<.001	0.544	0.688
	Item 3	0.624	0.034	18.504	<.001	0.558	0.690
	Item 4	0.578	0.033	17.607	<.001	0.514	0.643
*Renqing*	Item 5	0.608	0.048	12.669	<.001	0.514	0.702
	Item 6	0.600	0.050	12.112	<.001	0.503	0.697
	Item 7	0.597	0.048	12.421	<.001	0.503	0.691
*Xinren*	Item 8	0.729	0.037	19.741	<.001	0.657	0.801
	Item 9	0.803	0.039	20.676	<.001	0.727	0.879
	Item 10	0.738	0.038	19.530	<.001	0.664	0.812

**TABLE 2:** Second‐order factor loadings.

						95% confidence interval	
Factor	Indicator	Estimate	*SE*	*z*‐value	*p*	Lower	Upper
Second order	*Ganqing*	0.786	0.091	8.636	<.001	0.608	0.965
	*Renqing*	1.029	0.140	7.340	<.001	0.754	1.304
	*Xinren*	0.803	0.092	8.731	<.001	0.623	0.983

Based on Pearson's correlation matrix (see Table 3), strong relationships were found between all the variables of the present study, except for chronological age. The three *Guanxi* dimensions showed significant relationships among themselves and with organizational justice and contextual work performance.

**TABLE 3:** Descriptive statistics and Pearson's correlation matrix (*N* = 630)

Variable	M	*SD*	1	2		3		4		5	
1. Chronological age	48.25	7.16	—								
2. Organizational justice	3.21	1.24	0.062	—							
3. *Ganqing*	3.36	0.74	−0.057	0.424	***	—					
4. *Renqing*	2.98	0.83	0.059	0.529	***	0.386	***	—			
5. *Xinren*	3.98	0.87	0.015	0.481	***	0.351	***	0.452	***	—	
6. Contextual work performance	3.63	0.79	−0.001	0.341	***	0.500	***	0.361	***	0.344	***

*** *p <* .001.

### Mediation analyses

The mediation model tested the indirect effects of organizational justice on contextual work performance using JASP. This model examined the mediation roles of *Guanxi* dimensions, (*Ganqing*, *Renqing*, and *Xinren*). The global model predicted 30% of the variance on contextual work performance (*R* ^2^ = .300). On the one hand, the total direct effects of the organizational justice were not significant, as Table 4 showed, failing to provide support for H1. On the other, the total indirect effects were significant for all the dimensions, providing support for the hypotheses H2a–H2c, as can be seen in Table 4. The total effects of organizational justice on contextual work performance were statistically significant (Estimate = 0.274, *SE* = 0.030; *z*‐value = 9.112; *p* = .001, 95% CI [.215; .333]). The total indirect effects of organizational justice on contextual work performance were statistically significant (Estimate = 0.236, *SE* = 0.025; *z*‐value = 9.303; *p* = .001, 95% CI [.186; .285]).

**TABLE 4:** Direct and indirect effects of organizational justice on contextual performance

Effect	Estimate	Std. error	*z*‐value	*p*	95% confidence interval
Direct effects					
OJ → CWP	0.038	0.034	1.122	.262	[−0.029, 0.105]
Indirect effects					
OJ → Ganquing → CWP	0.131	0.017	7.680	<.001	[0.098, 0.165]
OJ → Renquing → CWP	0.055	0.018	3.083	.002	[0.020, 0.090]
OJ → Xinren → CWP	0.049	0.016	3.104	.002	[0.018, 0.080]

Abbreviations: CWP, contextual work performance; OJ, organizational justice; S.E., standard error.

The path coefficients for the relationships between organizational justice and the contextual work performance, as well as with the three *Guanxi* dimensions are displayed in Table 4.

### Moderated mediation analyses

Utilizing Hayes' Model 14, we explored the moderating influence of chronological age on the relationship between the three *Guanxi* dimensions (*Ganqing*, *Renqing*, and *Xinren*) and contextual work performance. The three *Guanxi* dimensions were entered at the same time in the model, conducting a parallel mediation analysis. For the *Ganqing* dimension, even though our model was significant (*F*(8,621) = 33.89, *p* < .001, *R* ^2^ = .304), the interaction between *Ganqing* and chronological age showed a non‐significant negative effect on contextual work performance (*B* = −0.060, *SE* = 0.089, 95% CI [−.234, .114], *p* = .499). This resulted in the non‐validation of hypothesis H3a. Notably, as chronological age rose, the bond between *Ganqing* and contextual work performance intensified for millennials (*B* = 0.116, *SE* = 0.0261, *p <* .001) but weakened for older workers (*B* = 0.101, *SE* = 0.0214, *p <* .001). A similar pattern was observed with the *Renqing* dimension: significant model (*F*(8,621) = 33.89, *p <* .001, *R* ^2^ = .304), yet the interaction between *Renqing* and chronological age yielded a non‐significant negative impact on contextual work performance (*B* = −0.123, *SE* = 0.086, 95% CI [−0.292, 0.046], *p* = .153). As age increased, the relationship between *Renqing* and contextual work performance became more prominent among millennials (*B* = 0.076, *SE* = 0.033, *p <* .001), while diminishing and losing significance for the older cohort (*B* = 0.032, *SE* = 0.018). Lastly, for the *Xinren* dimension, the model was significant (*F*(8,621) = 33.89, *p <* .001, *R* ^2^ = .304). The interaction between *Xinren* and chronological age had a non‐significant positive effect on contextual work performance (*B* = 0.078, *SE* = 0.082, 95% CI [−0.083, 0.239], *p* = .343). Interestingly, as age rose, the bond between *Xinren* and contextual work performance increased and became significant for older workers (*B* = 0.019, *SE* = 0.026, *p <* .001), while it diminished for millennials (*B* = 0.045, *SE* = 0.015), losing its statistical importance. The index of moderated mediation was not statistically significant in any case.

## DISCUSSION

The results of the present study reinforce and extend our understanding of the multifaceted relationships between organizational justice, *Guanxi* dimensions, and contextual work performance, while concurrently considering the potential moderating effect of chronological age (Farid et al., 2023). Our findings contribute important nuances to the literature and offer significant implications for organizational practices (Huang et al., 2023).

At the outset, the mediation analysis underscored the non‐significant direct effects of organizational justice on contextual work performance, thereby not supporting H1. However, significant indirect effects via the *Guanxi* dimensions offer a fresh perspective. This mirrors the research by X.‐P. Chen and C. C. Chen, which elucidated the centrality of *Guanxi* in business and organizational behaviors in the Chinese context (Chen & Chen, 2004). Given that *Guanxi* encompasses culturally embedded values and principles, such as mutual respect, trustworthiness, and reciprocal obligations (Barbalet, 2023), it is unsurprising that these dimensions mediate the relationship between perceptions of justice and consequent behaviors.

These findings reaffirm the argument posed by Farh et al. (1998), who contended that in collectivist cultures like China, interpersonal relationships (like *Guanxi*) play a more significant role in influencing organizational behaviors as compared to individualist cultures (Sheikh & Jamshed, 2023). When employees perceive fairness (organizational justice), it is likely that they lean into these ingrained cultural dimensions (*Ganqing*, *Renqing*, and *Xinren*) to manifest those perceptions in their performance (Farid et al., 2023), despite the fact that some negative correlates for *Guanxi* have been shown by authors (Au, 2022; He et al., 2023).

The nuanced age variations in this relationship are particularly intriguing. As Hofstede (2001) pointed out, the interplay of global influences and traditional values may manifest differently across generations (Wang et al., 2023). This could explain our findings where the influence of *Ganqing* on performance is accentuated among millennials but diminishes among older workers. Such a generational divergence aligns with the assertions of Zhou et al. (2011), who argued that younger Chinese workers, while still valuing traditional constructs like *Guanxi* (M. Li, Li, et al., 2023), might interpret and operationalize them differently in today's rapidly evolving workplace (Zhou et al., 2011). On the other hand, the strengthened bond between *Xinren* and performance among older workers possibly reflects the emphasis older generations place on trustworthiness, a value highlighted by Chen et al. (2013) as being of paramount importance in traditional Chinese business settings (Chen et al., 2013).

Furthermore, the findings provide deeper insights into the complex relationship between age, cultural dimensions, and workplace behaviors (Wang, 2023). This aligns with the work of Egri and Ralston (2004), who found generational differences in values and behaviors in a Chinese context, suggesting that while core cultural constructs remain stable, their manifestation and interpretation can differ across age groups (Egri & Ralston, 2004). At the same time, differential influences of peers' and social networks (Hao, Jiali, et al., 2023; Hao, Xin, et al., 2023) in social aspects (T. Yang & Zhang, 2024) can be observed when comparing urban and rural Chinese citizens (Fang et al., 2023) as well as locals with migrant Chinese people (Feng & Patulny, 2023; He et al., 2023).

In summary, this study illuminates the intricate dance between cultural values, perceptions of justice, age, and workplace behaviors in the Chinese context. It underscores the importance of understanding and appreciating these dynamics to foster optimal organizational outcomes. Future research could further explore these relationships across different cultural and organizational contexts (Chen et al., 2023; Zhou et al., 2017), providing a more comprehensive global perspective.

### Limitations of the present study

Despite the significant findings of the study, several limitations must be acknowledged. The study relied exclusively on online surveys to collect data, which, while offering extensive reach and convenience, might also introduce self‐selection bias. Individuals without internet access or those unfamiliar with online platforms could have been excluded, and the lack of face‐to‐face interactions might have led to ambiguities in responses. Moreover, the study's cross‐sectional nature provides a snapshot of the variables at a single point in time. Longitudinal studies are needed to explore this kind of process. As our sample was limited to Chinese employees, generalizing the findings to other cultural or geographical contexts should be approached with caution, given the pivotal role cultural differences play in organizational behavior (Wang & Yan, 2023). There might also be concerns regarding common method bias, given that all data were sourced from the same respondents via an online survey at the same time (T. Li et al., 2020; T. Li et al., 2022). Additionally, the binary categorization of age into millennials and older workers could oversimplify the effects of age on the variables studied. Reliance on self‐reported data brings potential biases, such as social desirability or recall biases, suggesting the utility of triangulating data in future research (Zheng & Yin, 2022; Zheng et al., 2022). Lastly, although the study employed established scales with known reliability and validity, interpretation differences might arise across diverse subgroups, especially when considering cultural contexts and translation nuances (Ahmed et al., 2013). As a result, while the study offers a foundational understanding of the relationships between organizational justice, *Guanxi* dimensions, chronological age, and contextual work performance, interpretations should be made cautiously.

### Suggestions for practitioners

Navigating the intricate nexus of organizational justice, *Guanxi* dimensions, and contextual work performance is no simple endeavor, particularly within the unique cultural milieu of China. This study's findings, while anchored in academic rigor, offer tangible pathways for practitioners. First, the indirect impact of organizational justice on contextual work performance, facilitated through *Guanxi* dimensions, underscores a pivotal managerial insight: While direct overtures of justice in the workplace are valuable, their potency is magnified when channeled through the cultural prism of *Guanxi*. This accentuates the need for organizational leaders to not only foster an environment replete with transparency, equity, and fairness but also to intertwine these principles with the culturally resonant dimensions of *Ganqing*, *Renqing*, and *Xinren* (Song & Jiang, 2022). In practical terms, initiatives aiming to bolster organizational justice should be nuanced with an understanding of the relational dynamics intrinsic to the Chinese workplace (H. Liu, Kong, et al., 2023).

Second, the differential impact of *Guanxi* dimensions based on chronological age offers another layer of complexity. As the age profile of the workforce evolves, so too should the strategies to harness the power of *Guanxi*. For millennials, emotional connections (*Ganqing*) and reciprocity (*Renqing*) appear more salient, necessitating a more relationally driven approach. For the older workforce, the dimension of trustworthiness (*Xinren*) gains prominence. Tailoring engagement and developmental initiatives with these nuances in mind can lead to enhanced contextual work performance (Wang et al., 2020).

In essence, practitioners stand to benefit immensely from a symbiotic fusion of organizational justice principles with the cultural wisdom of *Guanxi*, all the while being attuned to the generational idiosyncrasies that color these dynamics.

## CONCLUSION

This study illuminates the role of *Guanxi* dimensions in mediating the relationship between organizational justice and contextual work performance in a Chinese setting. While organizational justice alone did not directly impact work performance, its effect became significant when mediated by these cultural factors. The role of age as a moderator further complicates this relationship, showing variations between millennials and older workers. The contribution of the present study not only connects the Western approach to the traditional values of the Chinses society, it also offers valuable information that could be applied for Chinese organizations that lead with international counterparts, as well as for foreign firms that act in Chinese business environments.

## FUNDING INFORMATION

This work was supported in part by Liaoning Provincial Department of Education Project (LJKMZ20222009). This work was supported in part by Dalian Science and Technology Plan Project (2022JJ13FG101). This work was supported in part by Liaoning Social Science Planning Fund Project (L22ARK001). This work was supported in part by Liaoning Social Science Planning Fund Project (L22BGL007). This work was supported in part by Liaoning Provincial Education Science Planning Project (JG21DB040).

## CONFLICT OF INTEREST STATEMENT

The authors declare there are no conflicts of interest.

## ETHICS STATEMENT

The study was approved by the Ethics Committee of the Dalian Neusoft University of Information, ensuring that all procedures adhered to the stringent ethical standards set for academic research. The potential participants were also informed of their right to withdraw from the survey at any point should they choose to. Informed consent was obtained digitally before the commencement of the survey, guaranteeing their anonymity and confidentiality throughout the process.

Once participants completed the survey, they were thanked for their time and contribution. They were also provided with contact details of the research team in case they had any queries or sought feedback about the study.
